# “Make research more people‐centered”: Two ADNI participants share recommendations for more inclusive Alzheimer's disease research

**DOI:** 10.1002/alz.14070

**Published:** 2024-08-21

**Authors:** Sarah Walter, Rochelle Long, Cynthia Huling Hummel

**Affiliations:** ^1^ Alzheimer's Therapeutic Research Institute University of Southern California San Diego California USA; ^2^ Alzheimer's Clinical Trials Consortium (ACTC) Research Participant Advisory Board ATRI/USC San Diego California USA

We want to congratulate and thank the thousands of people who have given of their time and energy to gather and formulate the necessary data to study Alzheimer's disease. Over the past 20 years, more than 2400 participants have taken part in Alzheimer's Disease Neuroimaging Initiative (ADNI) at one of the many sites across North America. So much has been learned because of our willingness to step up and volunteer for cognitive testing, blood work, magnetic resonance imaging (MRI), positron emission tomography (PET) scans, and lumbar punctures. We are writing to share our experience as ADNI research participants and our recommendations for more inclusive research.

## 1 ROCHELLE'S STORY

I returned home to Cleveland 22 years ago to help with my mom who had fallen three times. I can honestly say that never in any scenario did I even think about research of any kind. But the day my aunt, who was living with us, was diagnosed, things changed. I immediately asked that they begin testing my mother. Within the year, she was diagnosed with early onset Alzheimer's. It was at one of our visits that the doctor asked if we'd be interested in participating in clinical trials. The response was an immediate, “Yes!” Especially since mom was now the fourth to be diagnosed in her family. All three of us began the journey into the world of clinical trials and research for Alzheimer's.

It was through the process of being both my mother and aunt's study partner, that my curiosity was piqued, and I volunteered to be a participant myself. Volunteering and working with the research team, I began to feel a real part of something bigger than I had ever imagined. I am trying daily to find a way to make living with this disease a little better for all of us, while contributing to research. In doing so, I also discovered both the lack and need for representation from underserved communities (especially my own of Black & Brown people). I began speaking at and volunteering for various events and programs, working closely alongside researchers. I have been reaching out to everyone I can, whether at home, work, or church, getting the word out for a desperate need for our community to participate. The research team made us feel valued not only for our contribution but as people who they cared about. I was at the office so frequently over the years that I actually felt like a member of the team.

I was approached about participating in the ADNI 3 study in 2019, just prior to the COVID‐19 shutdown in March of 2020. On the very day of the ordered shutdown, I was to have my first MRI and PET. This is when things really changed. I only heard from the office once a year when it was time to get my PET scan and MRI. This was a shock to me. I felt like I had lost my friends, people who understood what I was going through. It has been the loneliest study I've participated in during the past 20 years of research. I remember telling a friend it felt like “Wham, bam, and maybe thank you ma'am,” or “See ya, wouldn't want to be ya!”

I remember a particular incident where one of the techs at the hospital seemed annoyed at the questions I asked and her responses were quite curt. While the experience wasn't the fault of the research team, I didn't feel like I could call to complain, because the study got what they needed. Researchers need to understand you are dealing with people who have baggage. Baggage we can't see. It's not just how my community was treated in history; it's going on now. There is a stigma that science is only trying to get from us what they can get, so they can get the glory of what they have accomplished.

Relationships are so important in all facets of life. And a good relationship keeps growing.

### 1.1 My recommendations for researchers

- **Make research more people‐centered, not just disease‐centered**. We've been trying to establish relationships in one of our larger African American communities, because without those relationships, people are going to stay stuck on yesteryear, and won't start to trust. Ask participants for their feedback and fix the problems they tell you about!
- **Show participants they are valued beyond what the project is after**. Check in on your participants. A phone call, a card, or even an invitation to lunch could mean a lot.
- **Create a participant advisory board for ADNI and help your sites to set up community advisory boards**. My fire was reignited when I was invited to become a part of ACTC's Research Participant Advisory Board. I have met others who have lived experience and the same passion for research across the board. Studies need advice from people like us who are living with this disease.

### 1.2 Cynthia's story

I never intended to be involved in Alzheimer's research. Trust me, it wasn't on my bucket list. I was the pastor of a busy church in upstate New York, serving as a volunteer fire chaplain and singing in a country band. It was not on my radar—not even when my mom was diagnosed with Alzheimer's and no longer able to remember my name. That all changed when I started experiencing memory problems myself. I went from doctor to doctor seeking answers. I was diagnosed with amnestic mild cognitive impairment due to Alzheimer's and wasn't a surprise, but it was a sadness. I loved being a pastor. Leaving full‐time ministry was incredibly hard, but I had to, because I could no longer remember my parishioners or the stories that they had shared with me. I felt useless and I needed to do something that gave my life a sense of purpose. I called the Alzheimer's Association's TrialMatch and was matched with the ADNI study with my first visit in 2010.

Being a research participant was a wonderful way for me to channel my grief into something positive while helping to move science forward. I looked forward to my annual visits with my study team and their encouragement. I started reading everything I could find about Alzheimer's and dementia. I spoke at churches and at research conferences about my journey while urging others to step up and volunteer to be a research participant. It wasn't until I was invited to come to the Alzheimer's Association International Conference (AAIC) in 2021, that I truly understood the significance of ADNI and that the data are shared with researchers all over the world without embargo. ADNI has changed our understanding of Alzheimer's and other dementias. Now when I have my ADNI study lumbar punctures, I imagine my spinal fluid biomarkers, along with all the tubes of blood and the images of my brain, entered into a database and shared with researchers all over the world. It's amazing to think how many ADNI participants have stepped forward and how our data have led to treatments. It is humbling to think of being able to contribute to this amazing effort to change the narrative of Alzheimer's dementia from a death sentence to something “manageable” in the not‐too‐distant future. Volunteering in ADNI as a research participant has been both lifesaving and life‐giving. It gave me a new purpose and direction as a research ambassador and dementia advocate, and as an advisor on several national boards. I am grateful and feel blessed.

### 1.3 My recommendations for researchers

- **Return individual test results to participants**, including medical information such as biomarkers, MRIs and PET scan findings, and results from cognitive testing. It's true that some participants do not want this information, but many of us do want to know as we plan for our futures.^1^
- **Share ADNI findings with your participants and study partners on a regular basis**. Participants will be even more invested if they see that their participation in ADNI has led to a new discovery, understanding, and even treatment. It's something to celebrate. But don't be afraid to share the failures as well. Much can be learned from a project that fails.
- **Invite study participants to partner with you as research ambassadors**. If your participants feel the study is worthwhile, and that you are interested in their health and well‐being, they may be your best recruiters for ADNI and other studies.
- **Take time to support screen fails**. If someone does not meet the criteria for your ADNI or other study, take the time to thank them and refer them to another study where they might participate (TrialMatch 1‐800‐272‐2900). There are also many virtual studies that people might be able to join.

## 2 CONCLUDING THOUGHTS

In conclusion, we want to emphasize that ADNI is about teamwork: researchers, study participants, study partners, funders, and health care professionals all over the world. There can be no results—no new knowledge without the participants. We are doing our best to advance science! Together, we have and will continue to make a difference.

## CONFLICT OF INTEREST STATEMENT

The authors declare no conflicts of interest. Author disclosures are available in the Supporting information

## Supporting information

Supporting Information
